# Factor structure of psychosis screening questionnaire in Ugandan adults

**DOI:** 10.1186/s12888-023-05485-2

**Published:** 2024-01-09

**Authors:** Claire Kwagala, Amantia Ametaj, Hannah H. Kim, Joseph Kyebuzibwa, Rogers Okura, Anne Stevenson, Bizu Gelaye, Dickens Akena

**Affiliations:** 1 Department of Psychiatry, School of Medicine, College of Health Sciences, Makerere University, Kampala, Uganda; 2 Department of Epidemiology, Harvard T. H. Chan School of Public Health, Boston, MA USA; 3 Department of Social and Behavioral Sciences, Harvard T. H. Chan School of Public Health, Boston, MA USA; 4 Stanley Center for Psychiatric Research at Broad Institute of MIT and Harvard, Cambridge, MA USA; 5 The Chester M. Pierce, MD Division of Global Psychiatry, Massachusetts General Hospital, Boston, MA USA; 6 Department of Psychiatry, Faculty of Medicine and Health Sciences, Stellenbosch University, Cape Town, South Africa

**Keywords:** Africa, Psychosis screening questionnaire (PSQ), Psychometrics, Construct validity, Confirmatory Factor Analysis (CFA), Item response theory (IRT)

## Abstract

**Background:**

Psychotic disorders are common and contribute significantly to morbidity and mortality of people with psychiatric diseases. Therefore, early screening and detection may facilitate early intervention and reduce adverse outcomes. Screening tools that lay persons can administer are particularly beneficial in low resource settings. However, there is limited research evaluating the validity of psychosis screening instruments in Uganda. We aimed to assess the construct validity and psychometric properties of the Psychosis Screening Questionnaire (PSQ) in Uganda in a population with no history of a psychotic disorder.

**Methods:**

The sample consisted of 2101 Ugandan adults participating as controls in a larger multi-country case-control study on psychiatric genetics who were recruited between February 2018 and March 2020. Participants were individuals seeking outpatient general medical care, caretakers of individuals seeking care, and staff or students recruited from five medical facilities that were age 18 years or older and able to provide consent. Individuals were excluded who had acute levels of alcohol or substance use, including being under inpatient hospitalization or acute medical care for one of these conditions. We used confirmatory factor analysis (CFA) and item response theory (IRT) to evaluate the factor structure and item properties of the PSQ.

**Results:**

The overall prevalence screening positive for psychotic symptoms was 13.9% 95% CI (12.4,15.4). “Strange experiences” were the most endorsed symptoms 6.6% 95% CI (5.6,7.8). A unidimensional model seemed to be a good model or well-fitting based on fit indices including the root mean square error of approximation (RMSEA of 0.00), comparative fit index (CFI of 1.000), and Tucker-Lewis Index (TLI of 1.000). The most discriminating items along the latent construct of psychosis were items assessing thought disturbance followed by items assessing paranoia, with *a* parameter (discrimination) value of 2.53 and 2.40, respectively.

**Conclusion:**

The PSQ works well in Uganda as an initial screening tool for moderate to high-level of psychotic symptoms.

## Introduction

Psychotic disorders, including schizophrenia spectrum and bipolar affective disorders, are chronic severe mental illnesses that contribute significantly to high morbidity (years lived with disability) and mortality, mainly due to suicide risk [1]. Moreover, studies have demonstrated that people with psychotic disorders have an increased risk of cardiovascular disease [2], metabolic syndrome [3] and diabetes mellitus [4], which all predict premature mortality and can negatively impact quality of life [5].

The prevalence of psychotic disorders has been shown to vary widely worldwide with estimates between 0.8 and 31.4% [6]. However, many people have psychotic symptoms without a psychotic disorder and these too are associated with significant distress and impairment in personal, family, social, educational, occupational, and other important areas of life [7]. Research points to a higher prevalence of psychotic symptoms among individuals from low-resource settings [7]. For example, a study done in Dar-es-Salaam, Tanzania, showed the prevalence of psychotic symptoms to be 3.9% [8], and in another in a rural Kenyan setting, the prevalence of psychotic symptoms was found to be 8.1% [9].

Early screening for psychosis may facilitate early detection and prompt treatment for high-risk populations. In low-resource settings like Uganda, with a small number of mental health practitioners, screening tools that laypersons can administer are needed [10, 11]. Several instruments have been used to assess and screen for psychotic disorders, including self-report questionnaires, such as the Psychosis Screening Questionnaire (PSQ). Assessing the psychometric properties of PSQ in the Ugandan setting would help screen high-risk populations for psychosis, which could then be followed up with a diagnostic assessment.

The PSQ is preferred by clinicians and researchers because of its brevity and reliability and has been used with ethnic and cultural minorities in high-income countries. The PSQ was tested for equivalence in the United Kingdom in a study across five different ethnic groups in reporting psychotic symptoms [12]. It has also been adopted and used in Kenya [9], Tanzania [8] and Ethiopia [13].

There have been no studies in Uganda to date, to our knowledge, that examine the psychometric properties of the PSQ outside of our group. Our group has published a cross-cultural examination of the PSQ across Uganda, Ethiopia, Kenya, and South Africa [1]. However, this prior study was focused on a broad comparison of the scale’s performance across the four countries to test its equivalence across settings, without focusing on the specifics of the performance of PSQ from each country. The goal of this study was to examine the performance of PSQ in Uganda in depth, including fine grained analyses at the item level to understand its cultural relevance for the setting. Therefore, this study aimed to investigate the construct validity of the PSQ by exploring factor structure and item properties through item response theory analyses in Uganda with adults from a general medical setting.

## Method

We utilized data from the Neuropsychiatric Genetics of African Populations-Psychosis (NeuroGAP-Psychosis) study in Uganda, Ethiopia, Kenya, and South Africa. NeuroGAP-Psychosis is a case-control study aimed at expanding the understanding of genetic and environmental risk factors for psychotic disorders across different African populations, for the purposes of the current study, we analyzed data from Uganda only.

### Participants and study procedure

Study participants for the current study consisted of participants without psychosis (i.e., controls) in Uganda who were recruited between February 2018 and March 2020. Participants were individuals seeking outpatient general medical care, caretakers of individuals seeking care, and staff or students working at general medical facilities. Only controls were included in this study because patients with clinical diagnoses of psychosis (i.e., cases) were not administered the PSQ. Participants were recruited from the following medical facilities: Butabika National Mental Health Referral Hospital, Naguru, Arua, Mbarara, and Gulu Regional Referral Hospitals. Inclusion criteria for controls were age 18 years or older and able to provide consent. Individuals were excluded who had acute levels of alcohol or substance use, including being under inpatient hospitalization or acute medical care for one of these conditions. Ethical approval was obtained from all participating sites, including the Makerere University School of Medicine Research and Ethics Committee (SOMREC #REC REF 2016-057), the Uganda National Council for Science and Technology (UNCST #HS14ES), and the Harvard T.H. Chan School of Public Health (#IRB17-0822).

### Psychosis screening questionnaire

The presence of psychosis was assessed using the Psychosis Screening Questionnaire (PSQ), a self-reported brief screening instrument designed to detect psychotic symptoms. The PSQ has five primary (root) questions that assess the presence of psychotic symptoms: mania, thought-interference, paranoia, strange experiences, and hallucinations. Endorsement of any of the primary questions are followed by one to two secondary questions to further screen for psychotic experiences. The original PSQ assesses symptoms in the past year, but for our purposes we focused on lifetime symptoms “ever” in one’s life. We derived a binary response (0 = negative; 1 = positive) for each of the five psychotic symptoms based on responses to PSQ questions. In addition to the five binary responses, we derived a composite screening measure across the five symptoms. Presence of psychotic experiences was defined if positive on of any individual symptoms on the measure.

### Data analysis plan

Standard sociodemographic variables were collected, including age, sex at birth, level of education, marital status, and current living situation. Participant characteristics were described using means and standard deviation for continuous variables and counts and percentages for categorical variables. Prevalence estimates of psychotic symptoms were also calculated. All study participants from Uganda were included in all analyses, but three individuals from the total sample had missing data on the PSQ and were excluded from the below listed analyses.

### Confirmatory factor analysis

We examined the construct validity and factor structure of the PSQ by conducting confirmatory factor analysis (CFA) in Mplus 8 v.1.7. We tested a unidimensional factor structure based on past research and theory for the measure [14] including a study that reported one latent factor on a multi-ethnic British sample comparing PSQ’s equivalence across groups [12]. A split sample exploratory factor analysis was not possible due to a floor effect in our sample of controls with a low prevalence of psychotic disorders. Our model fit was calculated with a weighted least square mean and variance adjusted (WLSMV) estimator for categorical data, and measurement error was not assumed to be correlated among items.

CFA model fit was evaluated with the following goodness-of-fit metrics: (1) root mean square error of approximation (RMSEA) of 0.060 or below [15]; (2) comparative fit index (CFI) of 0.95 or above [15, 16]; and (3) Tucker-Lewis index (TLI) of 0.95 or above [15].

### Item response theory

The study further explored the factorial validity of the PSQ for Ugandan adults with item response theory (IRT) to better understand the relationship between the latent trait of psychosis and items on the PSQ. IRT accounts for how each item measures the latent construct and individual variation across the construct’s severity levels. IRT uses two main parameters, item discrimination and item difficulty, to describe the relationship between the participant, the latent construct (psychosis), and each PSQ item. The discrimination (or α) parameter describes the ability of each item to distinguish between degrees of psychotic symptom severity. The item difficulty (or *b*) indicates the location along the psychosis latent construct at which individuals have a ≥ 50% likelihood of endorsing a particular item.

We examined the assumptions required for an IRT model: unidimensionality, local independence, and monotonicity. The unidimensionality assumption was assessed by examining a one-factor CFA model, while the monotonicity was investigated via Mokken scaling analysis. After checking the assumptions, a 2-Parameter Logistic model was fitted using a unidimensional latent structure. Item information curves (IICs), item characteristic curves (ICCs), and the total information curves were generated using the R statistical program, version 3.6.2, packages Mokken and ltm.

## Results

### Demographic characteristics

The final analytic population consisted of 2,104 adults (Table 1, includes three individuals with missing data), with more participants who identified as female (56%) than male (44%). A large proportion of the participants were between the ages of 30 and 44 (40%), and a majority reported being married or cohabiting with a partner (53%). In addition, 42% reported attending at least some secondary school, and 32% reported finishing primary school.

**Table 1:** Participant demographics of Uganda (N = 2,104)*

	Count	%
Sex		
Female	1185	56.3
Male	919	43.7
Age categories in years (%)		
18–29	750	35.6
30–44	840	39.9
45–59	387	18.4
60+	127	6.0
Marital status (%)		
Single	600	28.5
Married or cohabitating	1110	52.8
Widowed	135	6.4
Divorced or separated	258	12.3
Level of education (%)		
No formal	114	5.4
Primary	663	31.5
Secondary	890	42.3
University	435	20.7
Living arrangements (%)		
Lives alone	324	15.4
Lives with parental family	357	17.0
Lives with spouse or partner	994	47.2
Lives with friends or other relatives	423	20.2

* *Note*: Counts may not add up to the total due to missing information for some participants

**Fig. 1:**
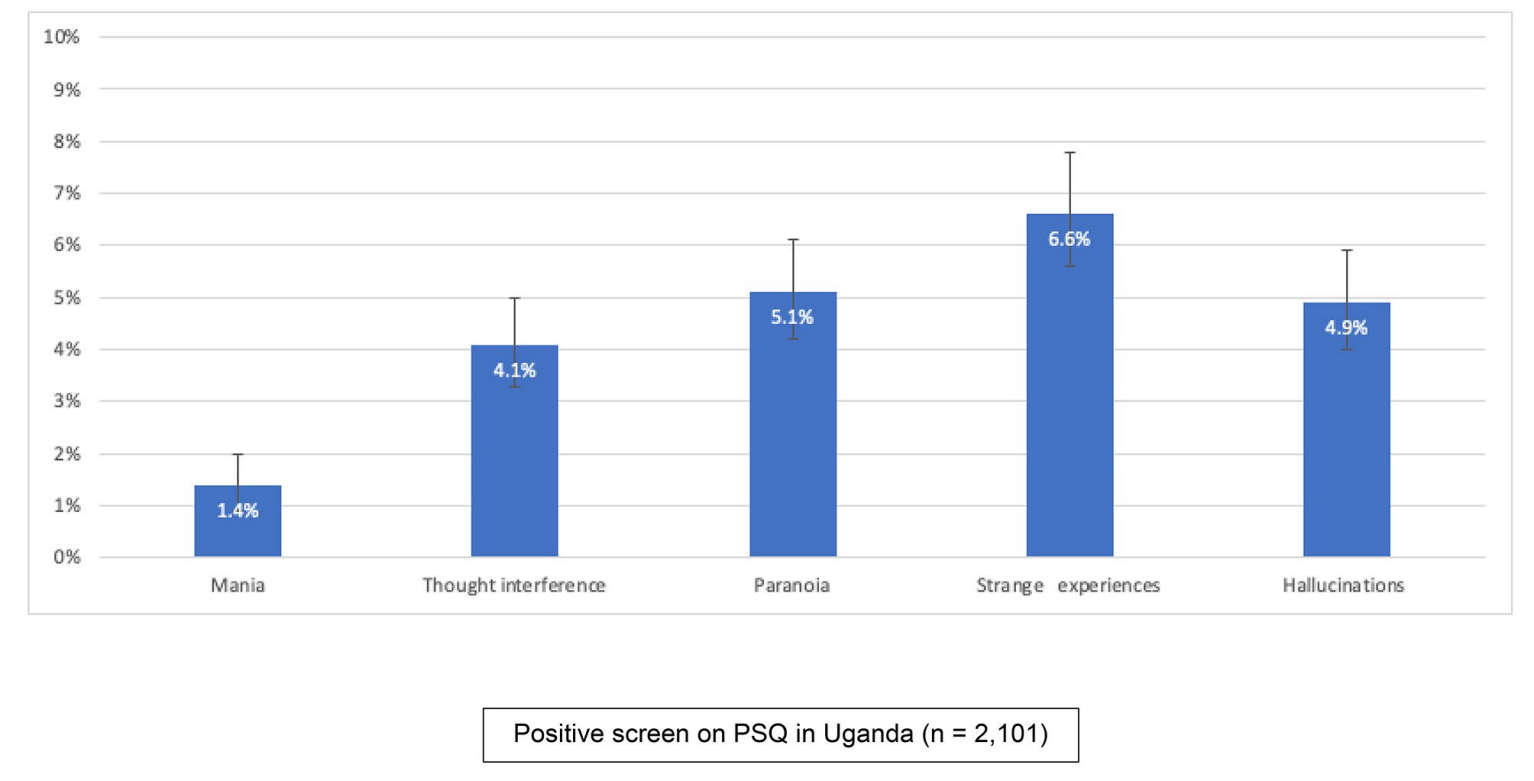
Prevalence of positive screen items on PSQ in Uganda (n = 2,101)

The overall prevalence of individuals screening positive for at least one psychotic symptom in their lifetime in our sample was 13.9% 95%CI (12.4, 15.4). Strange experiences was the most endorsed item 6.6% 95% CI (5.6, 7.8), followed by paranoia 5.1% 95%CI (4.2, 6.1), hallucinations 4.9% 95% CI (4.0, 5.9), and thought interference 4.1% 95% CI (3.3, 5.0). Mania was the least endorsed symptom 1.4% 95% CI (0.9, 2.0) (Fig. 1).

### Confirmatory factor analysis

We carried out a confirmatory factor analysis to examine the construct validity and factorial structure of the PSQ to assess model fit for a unidimensional model. The model fit indices and standardized factor loadings of each item are presented in Table 2. Indicators of goodness of fit, including RMSEA, CFI, and TLI indicate excellent fit (RMSEA of 0.00, TLI of 1.000 and CFI of 1.000) for a unidimensional model of the PSQ. The perfect model fit for categorical data was likely due to skewed binary indicators (i.e., floor effect) where we did not have sufficient power to reject the null model (Hu & Bentler, 1999; Methuen, 2014). Furthermore, all items loaded strongly (ranging from 0.72 to 0.79) on the unidimensional model of PSQ and provided additional evidence for a good model fit. The item with the weakest loading on the factor, although still strong, was mania (0.72).

**Table 2:** Model fit and parameter estimates for confirmatory factor analysis of PSQ in Uganda sample with and without mania items

	1-factor solution *with* the mania item N = 2,101*	
	Fit statistics:	
	χ^2^ [df, p] = 2.188 [8, 0.975]	
	RMSEA (90% CI) = 0.000 (0.000 to 0.017)	
	CFI = 1.000 TLI = 1.000	
	Model results:	
	Standardized factor loadings	(SE)
PSQ item:		
Thought interference	0.79	(0.04)
Paranoia	0.79	(0.04)
Strange experience	0.78	(0.04)
Hallucination	0.78	(0.04)
Mania	0.72	(0.06)

Note: RMSEA = root mean square error of approximation; CFI = comparative fit index; TLI = Tucker–Lewis index; SE = standard error

*3 participants were not included in the analysis because they had missing data on all PSQ items

### Item response theory

**Fig. 2:**
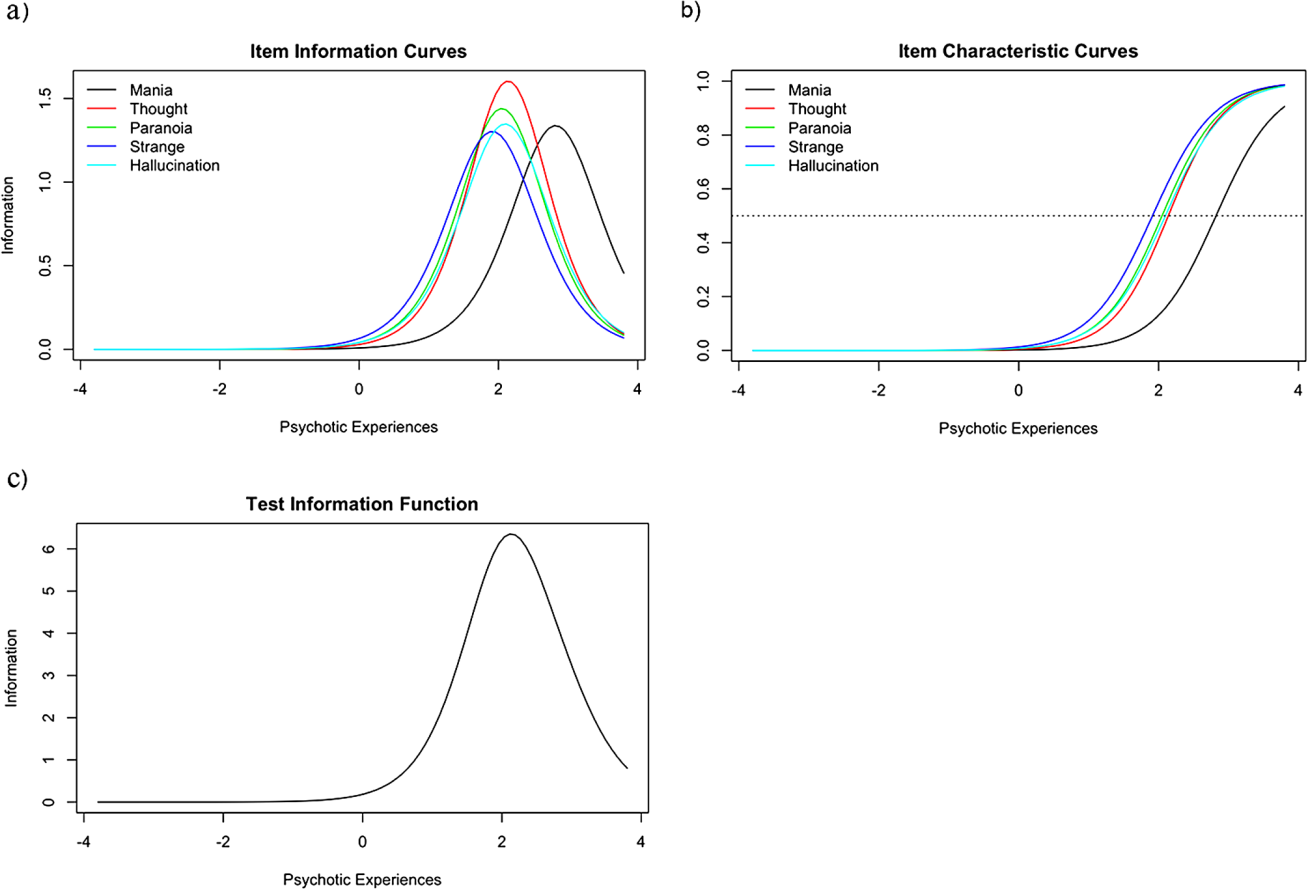
(**a**-**c**): Item Response Theory − **2a** (Item Characteristic Curves), **2b** (Item Information Curves, and **2c** (Test Information Function)

The IRT discrimination (α) and difficulty (*b*) parameters for each of the five PSQ items are presented in Table 3, and the IICs, ICCs, and test information function curves are found in Fig. 2. The most discriminating items along the latent construct were thought disturbance followed by paranoia, with *a* parameter (discrimination) values of 2.53 and 2.40, respectively. This is shown visually in the item information curves (Fig. 2a), where thought disturbance has the highest peak, followed by paranoia. Therefore, these two items (thought disturbance and paranoia) might best discriminate between participants with higher levels of psychotic experiences severity and participants with lower levels of psychotic experiences severity. The least discriminating item was strange experiences ($\alpha =2.53$), indicating that this item does not discriminate as effectively between participants at high and low levels of psychotic experiences relative to the other items. Overall, the items tend to discriminate between participants showing higher levels of psychotic experiences severity, given that the item information function peaks at the upper level of the latent construct (Fig. 2c). The test information function indicates that the PSQ is useful for screening moderate to high levels of psychotic symptoms rather than average or below-average levels.

**Table 3:** Item response theory PSQ parameters

Item	Difficulty (*b*)	Discrimination (α)
Mania	2.82	2.31
Thought	2.14	2.53
Paranoia	2.05	2.40
Strange	1.91	2.28
Hallucination	2.10	2.32

The ICCs visually represent the probability of endorsing items by the underlying construct severity (Fig. 2b). The ICC plot indicates that the probability of endorsing mania is highest when a person’s psychotic experience severity is high. In contrast, the other four items are similar in their probability of being endorsed at slightly moderate psychotic experiences levels, with strange experiences being the least difficult.

## Discussion

This study was one of the first to examine the psychometric properties of the PSQ in a Ugandan sample. The lifetime prevalence of psychotic symptoms in our sample was 13.9%, with strange experiences as the most endorsed item with a prevalence of (6.6%) and mania as the least endorsed symptom (1.4%). All items loaded strongly (ranging from 0.72 to 0.79) on a single psychosis latent factor measured by the PSQ and provided evidence for a good model fit. The IRT analysis indicated that the PSQ may provide more information for higher-than-average psychosis levels and that the PSQ will more likely identify individuals with a high level of psychosis compared to those with low levels.

Given that our study was one of the first of its kind in Uganda, the prevalence of lifetime psychotic symptoms of 13.9% found in our study cannot be compared with previous research in Uganda. The only other study in Uganda that used the PSQ outside of our reports from the NeuroGAP-Psychosis study noted a prevalence of 63% in first psychosis episode patients at Butabika National Psychiatric Referral Hospital [17]. However, this study does provide an adequate comparison since it was based on a retrospective chart review of people presenting for the first time specifically for mental health disorders while our study consisted of people with no history of psychotic disorders. In a study done in a Chinese adult population found a lifetime prevalence estimate of 5.5% for positive screens on the PSQ for the first phase of their study [18]. This is lower than the one found in our setting, and this could potentially be due to cultural differences and the different meanings attached to the different psychotic symptoms between the two settings.

On the confirmatory analysis, the PSQ performed well as a unidimensional construct. These findings are aligned with prior theory and research on the PSQ in high-income countries [7, 12]. IRT analyses showed that the most discriminating items along the latent construct were thought disturbance followed by paranoia, and these items gave the most precise information regarding psychosis. This finding is comparable to a study on psychotic symptoms in the United Kingdom examining diverse ethnic groups, which showed that paranoid symptoms were the most endorsed for participants of Caribbean ancestry [12].

Strange experiences, on the other hand, was the least precise symptom for identifying positive screens compared to other items. Given that the strange experiences item was the least discriminating item for psychosis and was the most endorsed, this item may not work well to screen for psychosis in Uganda. One explanation may be the description of a strange experience varies with different cultures in Uganda, with many experiences that would be considered strange being normalized due to the popular belief in supernatural experiences such as communication with the gods [19, 20]. Therefore, the prevalence proportion of 13.9% may have been overinflated by the strange experiences item.

Overall, the IRT analysis indicates that the PSQ provides valuable information about psychosis as a construct at higher levels of the latent trait, thus more accurately detecting moderate to severe levels of psychosis. The PSQ may be used by health care providers in busy clinics (due to its brevity) as well as by trained lay (community) health care workers to routinely screen for psychosis in primary health care facilities as well as community settings respectively. In low resourced settings, the PSQ provides a gateway to provision of care to persons with psychoses; screen positive case can always be referred to mental health care providers for a detailed diagnostic assessment. The measure may be less adept as a screen in generalist settings such as a screen administered to all primary care patients.

### Limitations

This study should be understood within its limitations. One major limitation is the lack of evaluation of measurement invariance analysis by key demographic (e.g., sex at birth) and clinical characteristics due to the low prevalence of psychotic experiences. Another limitation is the inability to conduct sub-group analyses due to few screen positive cases. The PSQ was not validated against a gold standard tool, this is a limitation of the design of our study given that the tool is from a neuropsychiatric and genetic study that aimed to reduce participant burden. Future research examining the PSQ against a clinical gold standard measure will shed further light on the measure’s utility in Uganda. In addition, future studies in this area may also consider using other self-report measures of psychosis to establish if the PSQ accurately measures the dimension of psychosis.

Recall bias was another important limitation in this study. This was again due to the study design that was aimed at capturing positive psychotic screens across the lifetime but that we are aware that most participants are likely reporting from more recent experiences.

We did not do rephrasing and item equivalence for “strange experiences” which was the least discriminating item. This is an important area of future research.

We did not use additional measures, such as the positive symptom scale of PANSS, to establish convergent and discriminant validity. We recommend that future studies in this area consider employing such measures to further validate the Psychotic Screening Questionnaire.

In this study, we only recruited participants in general hospital settings, and therefore the findings may not be generalizable to other populations. However, study strengths include administering and evaluating the PSQ in an understudied population using a large sample size.

## Conclusion

This is one of the first study to assess the psychometric properties of the Psychosis Screening Questionnaire in a Ugandan population to the best of our knowledge. This study estimated a lifetime prevalence of psychotic symptoms at 13.9% in a population with no history of a psychosis spectrum disorder, with strange experiences as the most endorsed symptom. Our findings show good construct validity and a one-dimensional structure for the PSQ in Uganda. The measure may be adept at screening individuals with higher levels of psychosis and be more helpful when administered to individuals displaying mental health symptoms for further diagnostic assessment.

### Recommendations

Future studies should validate the PSQ against gold standard measures such as the PANSS. Furthermore, there is need to examine the criterion validity of the PSQ in a large sample of participants so that sub-group analyses can be conducted.

## Data Availability

All data will be deposited and made available through the National Institute of Mental Health Data Archive at this site: https://nda.nih.gov/edit_collection.html?id=3805.
